# Synthesis of 2-Mercapto-(2-Oxoindolin-3-Ylidene)Acetonitriles from 3-(4-Chloro-5*H*-1,2,3-Dithiazol-5-Ylidene)Indolin-2-ones

**DOI:** 10.3390/molecules23061390

**Published:** 2018-06-08

**Authors:** Boris Letribot, Régis Delatouche, Hervé Rouillard, Antoine Bonnet, Jean-René Chérouvrier, Lisianne Domon, Thierry Besson, Valérie Thiéry

**Affiliations:** 1 University of La Rochelle, UMR CNRS 7266 LIENSs, 17000 La Rochelle, France; boris.letribot@gmail.com (B.L.); regis.delatouche@gmail.com (R.D.); herve.rouillard@hotmail.com (H.R.); antoine.bonnet@univ-lr.fr (A.B.); jean-rene.cherouvrier@univ-lr.fr (J.-R.C.); lisianne.domon@univ-lr.fr (L.D.); 2 Normandie University, UNIROUEN, INSA Rouen, CNRS, COBRA UMR 6014, 76000 Rouen, France; thierry.besson@univ-rouen.fr

**Keywords:** 1,2,3-dithiazole, Appel’s salt, 3-alkenyl-2-oxindole

## Abstract

Alkylidene oxindoles are important functional moieties and building blocks in pharmaceutical and synthetic chemistry. Our interest in biologically active compounds focused our studies on the synthesis of novel oxindoles, bearing on the exocyclic double bond at the C8, CN, and S groups. Extending the potential applications of Appel’s salt, we developed a new synthetic approach by investigating the reactions of C5-substituted 2-oxindoles with 4,5-dichloro-1,2,3-dithiazolium chloride (Appel’s salt) to give original (*Z*)-3-(4-chloro-5*H*-1,2,3-dithiazol-5-ylidene)indolin-2-one derivatives, and new 2-mercapto-(2-oxoindolin-3-ylidene)acetonitriles via a dithiazole ring-opening reaction. The work described in this article represents further applications of Appel’s salt in the conception of novel heterocyclic rings, in an effort to access original bioactive compounds. Fifteen new compounds were prepared and fully characterized.

## 1. Introduction

3-Alkenyl-2-oxindoles are an important class of heterocyclic compounds which are well established in the field of pharmaceutical chemistry, and they possess a large range of pharmacological activities [1,2,3,4,5,6]. For example, oxindole derivatives recently emerged as privileged scaffolds in the modulation of adenosine monophosphate-activated protein kinase (AMPK) [7], and 2-oxindole-based hydrazides are potent cytotoxic agents with apoptotic induction properties [8]. Substituted oxindoles are also very interesting building blocks for their role as starting materials toward more complex oxindole-based structures such as spirooxindoles [9,10]. Original piperidinoyl spirooxindoles can be obtained in very high yields and with excellent enantioselectivities via the hetero-Diels–Alder reaction between 2-aza-3-silyloxy-butadienes and alkylidene oxindoles [11]. It was also reported that donor or acceptor substituents at the ethylenic C8 carbon atom of isatylidenes constitutes an exceptional push–pull example, in which the electronic behavior of a fixed substituent on a double bond may be reversed by the electronic nature of the substituent at the opposite side of that double bond [12]. Palladium-catalyzed asymmetric allylic amination of racemic butadiene monoxide with oxindole derivatives was successfully developed using a chiral phosphoramidite–olefin hybrid ligand [13]. 

**Figure 1:**
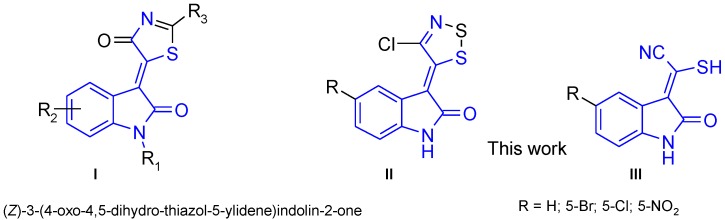
The (*Z*)-3-(4-oxo-4,5-dihydro-thiazol-5-ylidene)indolin-2-one pattern (**I**) [26,27], and synthesis targets 3-(4-chloro-5*H*-1,2,3-dithiazol-5-ylidene)indolin-2-one (**II**) and (2-oxoindolin-3-ylidene)-2-mercaptoacetonitrile (**III**).

**Scheme 1:**
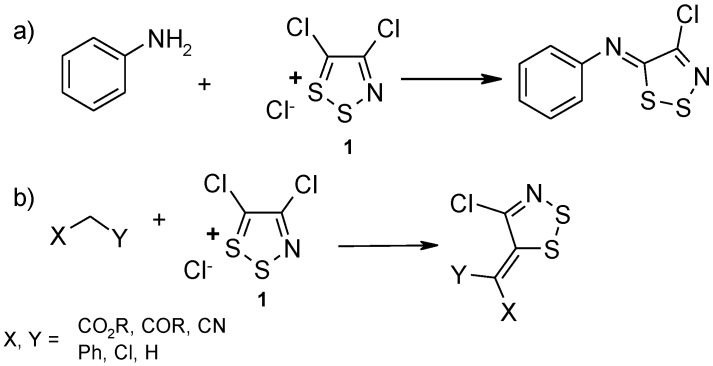
Reactivity of Appel’s salt with (**a**) N-arylamines; and (**b**) active methylene compounds.

Conventional synthetic routes to 3-alkenyl-oxindoles (aldol condensation of unsubstituted oxindoles or Wittig reactions on isatins) exhibit poor selectivity [14,15]. During the last decade, original synthetic routes based on intramolecular metallocatalyzed cyclizations leading to the formation of the C2–C3 or C3–C3a bonds of the 3-alkenyl-indolin-2-one moiety were established [16,17,18,19,20,21,22,23]. Development of new synthetic routes to these scaffolds remains a challenging task of current interest. Palmieri recently reported the synthesis of 3-alkylidene-2-oxindoles via the oxidation of sulfonyl indoles with NCS under flow chemical conditions [24]. The occurrence of the 3-alkenyl-oxindole skeleton in various natural and synthetic products generated the interest of many groups on account of its useful biological properties, and its versatility as a valuable intermediate in the synthesis of many compounds. As part of our ongoing research on oxindole derivatives, we previously designed, synthesized, and evaluated a new series of bis-oxindoles as potent kinase inhibitors [25]. In an effort to identify new pharmacological modulators of disease-relevant protein kinases with increased potency and selectivity, we launched a research program dealing with the synthesis of rare substituted 2-(2-oxoindolin-3-ylidene)acetonitrile derivatives bearing a sulfur atom directly attached to the external double bond. Nitrogen-containing heterocycles with sulfur atoms are an important class of compounds in medicinal chemistry. Few 3-(thiazol-5-ylidene)indoline-2-one derivatives (Figure 1, **I**) were reported in the literature [26,27]. To access the required 2-oxoindolylacetonitrile derivatives (Figure 1, **III**), we decided to explore a new route based on the use of 4,5-dichloro-1,2,3-dithiazolium chloride (Appel’s salt; Scheme 1, **1**) [28,29,30,31,32,33,34,35]. We planned on functionalizing the oxindole at the C3 position using Appel’s salt and commercially available oxindoles, and then, on studying the ring opening of original 3-(4-chloro-5*H*-1,2,3-dithiazol-5-ylidene)indolin-2-one (Figure 1, **II**) intermediates with various bases, such as sodium hydride and methyl magnesium bromide, or with triphenylphosphine as a nucleophile. In this work, we reported the synthesis of novel 3-(4-chloro-5*H*-1,2,3-dithiazol-5-ylidene)indolin-2-one (Figure 1, **II**) and (2-oxoindolin-3-ylidene)-2-mercaptoacetonitrile (Figure 1, **III**) derivatives.

## 2. Results and Discussion

It is well known that the reactive 5-chlorine atom of 4,5-dichloro-1,2,3-dithiazolium chloride (Scheme 1, **1**) can be displaced by a variety of nucleophiles, such as primary aromatic amines, or phenols, allowing access to *N*-heteroimino-1,2,3-dithiazole derivatives (Scheme 1, Path a) [36,37,38,39,40]. Several studies described the reactivity of Appel’s salt with active methylene compounds, using mainly malononitrile, tetracyanoethylene oxide (TCNEO), halo-substituted malononitriles and dimethylsulfonium dicyano-methylide, and malonate to afford 5-alkylidene-4-chloro-5*H*-1,2,3-dithiazole derivatives (Scheme 1, Path b) [41,42,43,44,45,46,47,48,49].

**Figure 2:**
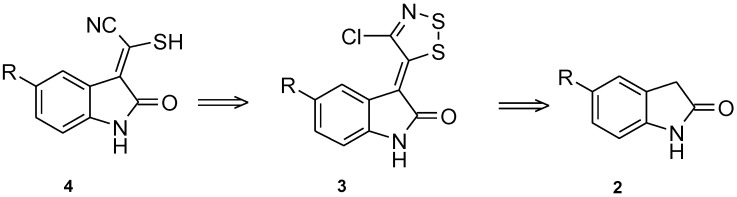
Retrosynthesis to 3-(4-chloro-5*H*-1,2,3-dithiazol-5-ylidene)indolin-2-one derivatives (**3**) and original (2-oxoindolin-3-ylidene)-2-mercaptoacetonitriles (**4**).

To generate the external double bond of the desired 3-alkenyl-indolin-2-one derivatives, we considered the electrophilic reactivity at the C1 position of Appel’s salt toward the active methylene at the C3 position of indolin-2-one derivatives (Figure 2, **2**).

**Scheme 2:**
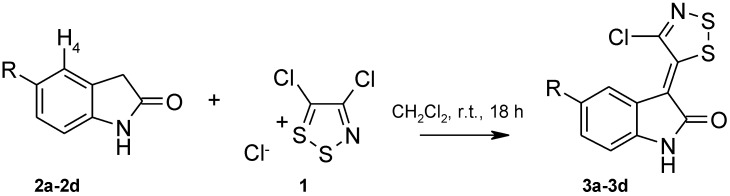
Synthesis of 3-(4-chloro-5*H*-1,2,3-dithiazol-5-ylidene)indolin-2-ones (**3a**–**3d**) via the condensation of 2-oxindoles (**2a**–**2d**) with Appel’s salt (**1**).

Performed at room temperature, the reactions between indolin-2-one derivatives (Scheme 2, **2a**–**d**) with a 1.1 equivalent of Appel’s salt (Scheme 2, **1**) in dichloromethane led to the corresponding (*Z*)-3-(1,2,3-dithiazol-5-ylidene)indololin-2-ones (Scheme 2, **3a**–**d**) in excellent yields (85–99%) (Scheme 2; Table 1). 

**Figure 3:**
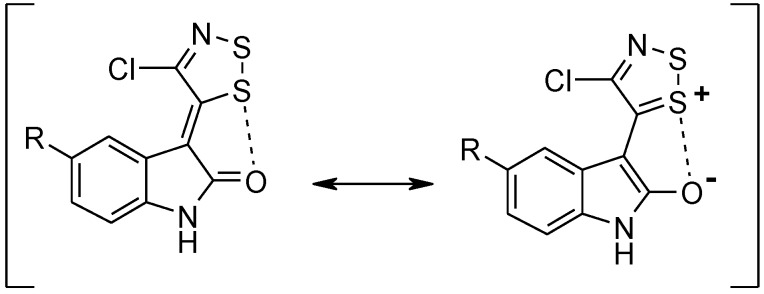
Representation of the O–S attractive interaction, and selected mesomer forms for the (*Z*)-3-(1,2,3-dithiazol-5-ylidene)indololin-2-ones (Table 1, **3a**).

From the ^1^H-NMR spectral data displayed, with only one set of aromatic signals in each case, it appeared that only one stereoisomer of each 3-(1,2,3-dithiazol-5-ylidene)indololin-2-one derivative (Table 1, **3a**–**3d**) was obtained. According to the work of Jeon et al. on several alpha-carbonylated 5-alkylidene-1,2,3-dithiazoles, we assumed that the (*Z*)-stereoisomers were obtained [45,46,47,48,49,50]. The stereoselectivity depends on steric hindrance between the chlorine atom at the C4 position of the dithiazole ring, and the oxygen atom on the carbonyl lactame of the indolin-2-one moiety. Additionally, an attractive interaction, already described by Rees et al., could also be established between this oxygen atom and the sulfur atom at the S1 position of the dithiazole moiety, in light of the mesomer forms (Figure 3) [45,46,47,48,49,50]. 

We noticed that the one-dimensional (1D) NMR spectra of 3-(1,2,3-dithiazol-5-ylidene)indolin-2-one (Table 1, **3a**–**3d**) displayed a strong downfield shift of the proton at the C4 position of the indolin-2-one core (8.19–9.13 ppm). Comparisons of the spectral data with those of the corresponding indolin-2-ones confirmed that the chemical shifts of this proton (H4) were displaced downfield by ~1 ppm, whereas the chemical shifts of the other aromatic protons were sensibly identical. This could be assigned to the proximity of the chlorine atom at the C4 position of the dithiazole ring. Similar observations were mentioned in the literature for (*Z*)-5-(2-oxoindolin-3-ylidene)thiazolidin-4-ones (Figure 1, **I**) [26]. In these cases, X-ray diffraction analysis probed that the C=O of the thiazolidinone moiety interacted with the proton at the C4 position of the indolin-2-one core, causing a strong downfield shift of the proton in proximity [26,27].

### Dithiazole Ring Opening

The ring-opening chemical property of the dithiazole moiety relies on its ability to undergo nucleophilic attacks, thanks to the latent cyano group within the dithiazole moiety. This was widely exploited with *N*-arylimino-1,2,3-dithiazoles to generate heterocycles functionalized with sulfur atoms and a carbonitrile [51,52,53,54,55,56,57]. To date, the reactivity of 5-alkylidene-1,2,3-dithiazoles is rarely explored with triphenylphosphine [58], and is never described with strong bases. By analogy with our previous work on *N*-arylimino-1,2,3-dithiazole ring opening via nucleophilic attack at S1 and/or S2, we expected to obtain 3-alkenyl-oxindoles functionalized by a thiol and a carbonitrile function (Figure 1, **III**) [59,60]. 

**Scheme 3:**
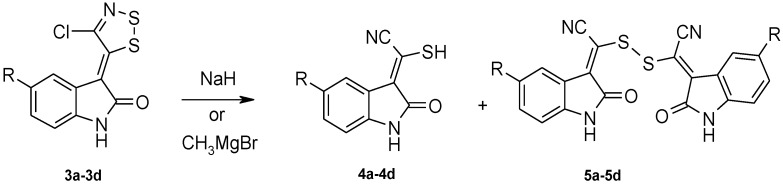
Dithiazole ring opening with sodium hydride or methyl magnesium bromide. Conversion of 3-(1,2,3-dithiazol-5-ylidene)indololin-2-ones (**3a**–**d**) into 2-mercapto-2-(2-oxoindolin-3-ylidene)acetonitriles (**4a**–**d**) and 2,2′-disulfanediylbis(2-(2-oxoindolin-3-ylidene)acetonitriles) (**5a**–**5b**).

We previously showed that attack of some *N*-arylimino-1,2,3-dithiazoles with sodium hydride or methyl magnesium bromide led, in each case, to one product: *N*-aryl isothiocyanate and cyanothioformamide [59,60]. Treatment of 3-(1,2,3-dithiazolylidene)indololin-2-ones (Scheme 3, **3a**–**3d**) by two equivalents of sodium hydride or methyl magnesium bromide in tetrahydrofuran led to the expected 2-mercapto-2-(2-oxoindolin-3-ylidene)acetonitrile derivatives (Scheme 3, **4a**–**4d**) with modest to good yields (23–88%) (Scheme 3; Table 2 and Table 3). It must be noted that sodium hydride (49–88%) was more efficient than methyl magnesium bromide (0–33%).

The ring opening of dithiazoles (Scheme 3, **3a**–**3c**) with NaH afforded the expected dithiazoles (Scheme 3, **4a**–**4c**), accompanied by a disulfur dimer as a side product (Scheme 3, **5a**–**5c**). The reaction with methyl magnesium bromide led only to dimers **5a** and **5b** (Table 2 and Table 3). Whatever the base, no traces of the nitro dimer (Table 2 and Table 3, **3d**) were detected. The formation of these dimers seemed to be independent of the nature of the base used, suggesting a preponderant role of substituents on the aromatic ring.

The assignment of stereochemistry to the dithiazoles, and the comparison of chemical shifts of ^1^H-NMR of H4 of the dithiazole precusors (Table 2, **3a**–**3d**) and mercaptoacetonitrile derivatives (Table 2, **4a**–**4d**) showed that the (*Z*) isomer was obtained (Table 3). 

**Scheme 4:**
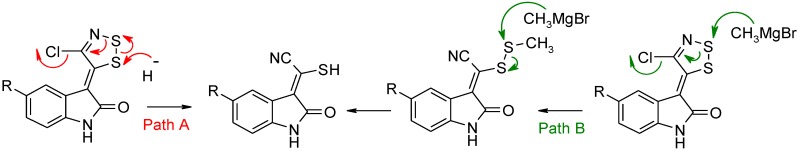
Proposed mechanisms according to the use of NaH (Path A) or methyl magnesium bromide (Path B).

According to the nature of the base used, it seems probable that, with sodium hydride, the reduction started via an attack on sulfur S1, leading to dithiazole ring opening followed by the release of sulfur (^1^/_8_ S_8_) and the formation of the latent cyano group (Scheme 4, Path A). In Path B of Scheme 4, with methyl magnesium bromide, the products obtained could only be formed via an attack of the carbanion on the sulfur atom S2. This attack was followed by the generation of the carbonitrile. The 2-mercapto-2-(2-oxoindolin-3-ylidene)acetonitriles (Table 3, **4a**–**d**) were finally obtained after the attack of a second carbanion on the sulfur atom S2, accompanied by the release of dimethyl sulfide (Scheme 4, Path B).

**Scheme 5:**
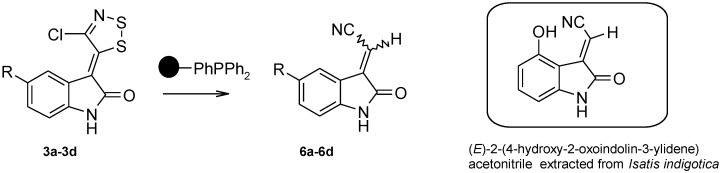
Synthesis of 2-(2-oxoindolin-3-ylidene) derivatives (**6a**–**6d**) via the ring opening of alkenyldithiazole (**3a**–**3d**) with supported triphenylphosphine.

5-Alkylidene-1,2,3-dithiazoles (Scheme 5, Table 4, **3a**–**3d**) were then treated with two equivalents of supported triphenylphosphine as a nucleophile in dichloromethane. Dithiazoles (Scheme 5, Table 4, **3a**–**3d**) were directly converted into 2-(2-oxoindolin-3-ylidene)acetonitriles (Scheme 5, Table 4, **6a**–**6d**). Whatever the experimental conditions, unexpected open products bearing no sulfur atoms were always obtained in modest to good yields (27–82%), with no trace of mercaptoacetonitriles being isolated (Scheme 4). A mixture of the two stereoisomers was obtained with the (E) isomer as the main compound for **6b**–**6d** (Table 4). A substituent effect can be supposed since the 5-substituted oxindole-dithiazoles (Table 4, **3b**–**3d)** afforded mainly the (E) isomer and the nude isomer, while **6a** (Table 4) afforded mainly the (*Z)* isomer.

The stereochemistry of the products (Table 4, **6b**–**6d**) was unambiguously determined using ^1^H-NMR, and through comparison with data from the literature [60,61]. Osman et al. previously reported the synthesis and the stereochemistry of 2-(2-oxoindolin-3-ylidene)acetonitrile isomers (Table 4, **6a**) via a Wittig reaction between an isatin and a phosphorus ylide [61]. The stereochemistry of a number of functionally substituted 2-oxoindolin-3-ylidene derivatives and substituted acetonitriles was established by Ross [60].

It is interesting to note that these derivatives are analogues of the natural product (E)-2-(4-hydroxy-2-oxoindolin-3-ylidene)acetonitrile, extracted from the plant *Isatis indigotica* (Scheme 5) [62].

## 3. Materials and Methods

### 3.1. General Information

All commercially available chemicals and solvents were purchased from commercial sources, and used without further purification. 2-Oxindoles (Table 1, 2-oxindole **2a**, 5-bromo-2-oxindole **2b**, 5-chloro-2-oxindole **2c**, and 5-nitro-2-oxindole **2d**) were purchased from Sigma-Aldrich (Saint Quentin Fallavier, France). Anhydrous THF was purchased from Sigma-Aldrich, and was stored over 4 Å molecular sieves. All other solvents used were analytical grade purity. All reactions were carried in an inert atmosphere of argon, and were monitored by thin layer chromatography using Merck silica gel plates (60 F_254_/0.2 mm thickness). Purification through flash chromatography was performed using Merck silica gel 60 (63–200 μm; 70–230 mesh). ^1^H- and ^13^C-NMR spectra were recorded on a JEOL JNM-LA400 (Croissy Sur Seine, France) operating at 400 MHz (^13^C 100 MHz) with tetramethylsilane as an internal standard in CDCl_3_, acetone-*d*_6_, or DMSO-*d*_6_ solvent. Chemical shifts were given in ppm (δ), and coupling constants (*J*) were reported in Hertz. Proton-coupling patterns were described as singlet (s), doublet (d), triplet (t), or multiplet (m). High-resolution mass spectra (HRMS) were recorded on a Waters Q-TOF 2 (CRMPO, University of Rennes) or Waters Q-TOF instrument (CCA, University of La Rochelle). Melting points were determined in open capillaries on a Stuart melting point SMP3 digital melting point apparatus, and were uncorrected. IR spectra were recorded on a Perkin-Elmer Spectrum 100 IRTF instrument (Courtaboeuf-Les Ulis, France), and values were expressed in cm^−1^.

### 3.2. Synthesis

#### 3.2.1. General Procedure for the Synthesis of 3-(4-Chloro-5*H*-1,2,3-Dithiazol-5-Ylidene)Indolin-2-one Derivatives (Table 2, **3a**–**d**).

Appel’s salt (Scheme 2, **1**; 4,5-dichloro-1,2,3-dithiazolium chloride; 15.76 mmol) was added to a solution of each appropriate 2-oxindole derivative (Scheme 2, **2a**–**d**; 14.33 mmol) in CH_2_Cl_2_ (20 mL). After a few minutes, the uncolored mixture turned to a dark red color, and was stirred at room temperature for 18 h. The solvent was evaporated under reduced pressure, and the crude product was dissolved with acetone (a minimum of 30 mL), and then precipitated with water (300 mL). The heterogeneous mixture was filtered in a vacuum, and the red solid was washed three times with water (25 mL). This solid was finally dried over phosphorus pentoxide in a vacuum in a desiccator.

*(Z)-3-(4-Chloro-5H-1,2,3-dithiazol-5-ylidene)indolin-2-one* (Table 2, **3a**). Yield: 96%. Red solid; melting point (m.p.): 271–273 °C. ν_max_ (cm^−1^): 3244, 1693, 1613, 1462, 1336, 1221, 1203, 1080, 743. ^1^H-NMR (400 MHz, DMSO-*d*_6_) δ = 11.22 (*s*, 1H), 8.19 (*d*, *J* = 8.0 Hz, 1H), 7.17 (*t*, *J* = 7.9 Hz, 1H), 6.91–6.97 (*m*, 2H). ^13^C-NMR (400 MHz, DMSO-*d*_6_) δ = 169.5, 149.0, 143.8, 139.8, 127.4, 126.5, 120.8, 120.1, 115.7, 110.2. HRMS (ES) *m*/*z* calculated for C_10_H_5_ClN_2_OS_2_ [M + H]^+^ 267.9538, found 267.9545.

*(Z)-5-Bromo-3-(4-chloro-5H-1,2,3-dithiazol-5-ylidene)indolin-2-one* (Table 2, **3b**). Yield: 96%. Red solid; m.p.: 222–224°C. ν_max_ (cm^−1^): 3147, 1665, 1641, 1612, 1485, 1454, 1427, 1317, 1246, 1092, 915, 803. ^1^H-NMR (400 MHz, DMSO-*d*_6_) δ = 11.40 (*s*, 1H), 8.36 (*d*, *J* = 1.6 Hz, 1H), 7.41 (*dd*, *J* = 8.3, 1.7 Hz, 1H), 6.97 (*d*, *J* = 8.3 Hz, 1H). ^13^C-NMR (400 MHz, DMSO-*d*_6_) δ = 169.4, 151.1, 143.9, 138.7, 129.5, 128.4, 122.1, 113.9, 112.6, 112.0. HRMS (ES) *m*/*z* calculated for C_10_H_4_BrClN_2_OS_2_ [M + H]^+^ 346.8715, found 346.8715.

*(Z)-5-Chloro-3-(4-chloro-5H-1,2,3-dithiazol-5-ylidene)indolin-2-one* (Table 2, **3c**). Yield: 99%. Red solid; m.p.: 220–222 °C. ν_max_ (cm^−1^): 3148, 1658, 1639, 1614, 1456, 1427, 1318, 1244, 923, 812, 804. ^1^H-NMR (400 MHz, DMSO-*d*_6_) δ = 11.40 (*s*, 1H), 8.24 (*d*, *J* = 2.0 Hz, 1H), 7.29 (*dd*, *J* = 8.3, 2.1 Hz, 1H), 7.02 (*d*, *J* = 8.3 Hz, 1H). ^13^C-NMR (400 MHz, DMSO-*d*_6_) δ = 169.5, 151.1, 143.9, 138.4, 126.7, 125.7, 124.8, 121.6, 114.1, 111.5. HRMS (ES) *m*/*z* calculated for C_10_H_4_Cl_2_N_2_OS_2_ [M − H]^−^ 300.9064, found 300.9063.

*(Z)-3-(4-Chloro-5H-1,2,3-dithiazol-5-ylidene)-5-nitroindolin-2-one* (Table 2, **3d**). Yield: 85%. Red solid; m.p.: 252–254 °C. ν_max_ (cm^−1^): 3381, 3165, 3085, 3024, 1653, 1616, 1515, 1441, 1333, 1301, 1240, 1133, 830, 692, 686, 645. ^1^H-NMR (400 MHz, DMSO-*d*_6_) δ = 11.92 (*s*, 1H), 9.13 (*s*, 1H), 8.16 (*d*, *J* = 8.8 Hz, 1H), 7.16 (*d*, *J* = 8.8 Hz, 1H). ^13^C-NMR (400 MHz, DMSO-*d*_6_) δ = 167.0, 152.8, 144.5, 143.7, 141.3, 122.9, 121.3, 120.1, 112.7, 110.1. HRMS (ES) *m*/*z* calculated for C_10_H_4_ClN_3_O_3_S_2_ [M − H]^−^ 311.9304 found 311.9304.

#### 3.2.2. General Procedure for the Synthesis of 2-Mercapto-2-(2-Oxoindolin-3-Ylidene)Acetonitrile Derivatives (Table 3, **4a**–**4d**)

##### *Path* *A* *with* *NaH*

Sodium hydride at 60% in mineral oil (12 mmol) was added portionwise to a solution of each appropriate 5-alkylidene-1,2,3-dithiazole (Scheme 3, **3a**–**d**; 6 mmol) in dry THF (15 mL). The dark red solution turned to dark yellow color after total consumption of the starting material. The solvent was evaporated under reduced pressure, and the crude product was quenched with brine (75 mL) and ethyl acetate (20 mL), before being extracted three times with ethyl acetate (200 mL). The organic layer was washed with brine, dried over magnesium sulfate, filtered, and concentrated under reduced pressure. The crude product was purified through flash chromatography on a silica gel column (100% ethyl acetate).

##### *Path* *B* *with* *CH_3_MgBr*

Methyl magnesium bromine at 3 mol·L^−1^ (1.2 mmol) was added portionwise to a solution of each appropriate 5-alkylidene-1,2,3-dithiazole (Scheme 3, **3a**–**d**; 0.6 mmol) in dry THF (4 mL). The dark red solution turned to a dark yellow color after total consumption of the starting material. The solvent was evaporated under reduced pressure, and the crude product was quenched with brine (15 mL) and ethyl acetate (10 mL), before being extracted three times with ethyl acetate (100 mL). The organic layer was washed with brine, dried over magnesium sulfate, filtered, and concentrated under reduced pressure. The crude product was purified through flash chromatography on a silica gel column (100% ethyl acetate).

*(Z)-2-Mercapto-2-(2-oxoindolin-3-ylidene)acetonitrile* (Table 3, **4a**). Yield**:** 49% (*Path A*), 33% (*Path B*). Dark Solid; m.p.: 295–297 °C. ν_max_ (cm^−1^): 3239, 2207 (CN), 1647, 1616, 1509, 1458, 1332, 1185, 1086, 777, 724. ^1^H-NMR (400 MHz, acetone-*d*_6_) δ = 9.09 (*s*, 1H), 8.99 (*d*, *J* = 7.5 Hz, 1H), 7.03 (*t*, *J* = 7.4 Hz, 1H), 6.88 (*t*, *J* = 7.6 Hz, 1H), 6.81 (*d*, *J* = 7.7 Hz, 1H). ^13^C-NMR (400 MHz, acetone-*d*_6_) δ = 168.0, 158.0, 138.7, 134.1, 126.1, 123.6, 121.2, 120.9, 118.0, 108.7. HRMS (ES) *m*/*z* calculated for C_10_H_6_N_2_OS [M + Na]^+^ 225.0098, found 225.0098.

*(Z)-2-(5-Bromo-2-oxoindolin-3-ylidene)-2-mercaptoacetonitrile* (Table 3, **4b**). Yield: 66% (*Path A*), 25% (*Path B*). Dark solid; m.p.: 330–332 °C. ν_max_ (cm^−1^): 3275, 2217 (CN), 1646, 1610, 1508, 1457, 1443, 1303, 1192, 1175, 1089, 800. ^1^H-NMR (400 MHz, acetone-*d*_6_) δ = 9.30 (*s*, 1H), 9.23 (*d*, *J* = 2.1 Hz, 1H), 7.18 (*dd*, *J* = 8.2, 2.2 Hz, 1H), 6.81 (*d*, *J* = 8.2 Hz, 1H). ^13^C-NMR (400 MHz, acetone-*d*_6_) δ = 167.7, 151.6, 139.3, 137.3, 133.3, 129.4, 128.3, 125.4, 124.9, 110.6. HRMS (ES) *m*/*z* calculated for C_10_H_5_BrN_2_OS [M + Na]^+^ 302.9204, found 302.9205.

*(Z)-2-(5-Chloro-2-oxoindolin-3-ylidene)-2-mercaptoacetonitrile* (Table 3, **4c**). Yield: 68% (*Path A*). Dark solid; m.p. > 350 °C. ν_max_ (cm^−1^): 3276, 2209 (CN), 1642, 1616, 1517, 1449, 1302, 1193, 1177, 1091, 800. ^1^H-NMR (400 MHz, acetone-*d*_6_) δ = 9.69 (*s*, 1H), 9.55 (*d*, *J* = 2.3 Hz, 1H), 7.48 (*dd*, *J* = 8.1, 2.2 Hz, 1H), 7.29 (*d*, *J* = 8.2 Hz, 1H). ^13^C-NMR (400 MHz, acetone-*d*_6_) δ = 167.8, 152.0, 137.0, 129.5, 126.4, 125.4, 125.0, 122.5, 120.3, 110.0. HRMS (ES) *m*/*z* calculated for C_10_H_5_ClN_2_OS [M − H]^−^ 234.9733, found 234.9733. 

*(Z)-2-Mercapto-2-(5-nitro-2-oxoindolin-3-ylidene)acetonitrile* (Table 3, **4d**). Yield: 88% (*Path A*). Dark solid; m.p. > 350 °C. ν_max_ (cm^−1^): 3310, 2211 (CN), 1655, 1617, 1586, 1508, 1447, 1406, 1332, 1288, 1057, 986, 877, 748. ^1^H-NMR (400 MHz, acetone-*d*_6_) δ = 9.91 (*d*, *J* = 2.4 Hz, 1H), 9.82 (*s*, 1H), 8.03 (*dd*, *J* = 8.5, 2.4 Hz, 1H), 7.02 (*d*, *J* = 8.6 Hz, 1H). ^13^C-NMR (400 MHz, acetone-*d*_6_) δ = 168.0, 153.9, 143.8, 143.1, 128.2, 124.0, 122.5, 119.8, 117.6, 108.4. HRMS (ES) *m*/*z* calculated for C_10_H_5_N_3_O_3_S [M + H]^+^ 248.0130, found 248.0128.

*2,2′-Disulfanediylbis(2-(2-oxoindolin-3-ylidene)acetonitrile)* (Table 3, **5a**). Yield: 46% (*Path A*), 30% (*Path B*). Dark Solid; m.p.: 295–297 °C. ν_max_ (cm^−1^): 3239, 2207 (CN), 1647, 1616, 1509, 1458, 1332, 1185, 1086, 777, 724. ^1^H-NMR (400 MHz, acetone-*d*_6_) δ = 9.15 (*s*, 2H), 8.10 (*d*, *J* = 7.8 Hz, 2H), 7.03 (*t*, *J* = 7.7 Hz, 2H), 6.90 (*t*, *J* = 7.5 Hz, 2H), 6.85 (*d*, *J* = 7.5 Hz, 2H). HRMS (ES) *m*/*z* calculated for C_20_H_10_N_4_O_2_S_2_ [M + Na]^+^ 425.0143, found 425.0142.

*2,2′-Disulfanediylbis(2-(5-bromo-2-oxoindolin-3-ylidene)acetonitrile)* (Table 3, **5b**). Yield: 33% (*Path A*), 5% (*Path B*). Dark solid; m.p. > 350°C. ν_max_ (cm^−1^): 3275, 2217 (CN), 1646, 1610, 1508, 1457, 1443, 1303, 1192, 1175, 1089, 800. ^1^H-NMR (400 MHz, DMSO-*d*_6_) δ = 10.06 (*s*, 2H), 9.00 (*d*, *J* = 2.1 Hz, 2H), 7.17 (*dd*, *J* = 8.3, 2.0 Hz, 2H), 6.65 (*d*, *J* = 8.0 Hz, 2H). ^13^C-NMR (400 MHz, DMSO-*d*_6_) δ = 165.79, 147.28, 137.57, 128.59, 127.68, 124.54, 123.89, 118.79, 111.88, 109.50. HRMS (ES) *m*/*z* calculated for C_20_H_8_Br_2_N_4_O_2_S_2_ [M + Na]^+^ 580.8353, found 580.8343. 

*2,2′-Disulfanediylbis(2-(5-chloro-2-oxoindolin-3-ylidene)acetonitrile)* (Table 3, **5c**). Yield: 30% (*Path A*). Dark solid; m.p. > 350 °C. ν_max_ (cm^−1^): 3276, 2209 (CN), 1642, 1616, 1517, 1449, 1302, 1193, 1177, 1091, 800. ^1^H-NMR (400 MHz, DMSO-*d*_6_) δ (ppm): 10.05 (*s*, 2H), 8.86 (*d*, *J* = 2.1 Hz, 2H), 7.04 (*dd*, *J* = 8.1, 2.2 Hz, 2H), 6.69 (*d*, *J* = 8.1 Hz, 2H). HRMS (ES) *m*/*z* calculated for C_20_H_8_Cl_2_N_4_O_2_S_2_ [M + Na]^+^ 492.9363, found 492.9357.

#### 3.2.3. General Procedure for the Synthesis of 2-(2-Oxoindolin-3-Ylidene)Acetonitrile Derivatives (Table 4, **6a**–**d**)

Supported triphenylphosphine at 2 mmol·g^−1^ (1.04 mmol) was added to a solution of each appropriate 5-alkylidene-1,2,3-dithiazole (Scheme 5, **3a**–**d**; 0.52 mmol) in CH_2_Cl_2_ (5 mL). The mixture was stirred at room temperature for 24 h, and filtered in a vacuum. The resin was washed three times with ethyl acetate (20 mL). The organic layer was washed with brine, dried over magnesium sulfate, filtered, and concentrated under reduced pressure. The crude product was purified through flash chromatography on a silica gel column (ethyl acetate/petroleum ether).

*2-(2-Oxoindolin-3-ylidene)acetonitrile* (Table 4, **6a**) [61]. Yield: 88%. Orange solid; m.p.: 173–175 °C. (*E*): ν_max_ (cm^−1^): 3192, 2218 (CN), 1715, 1610, 1463, 1353, 1318, 1216, 1150, 778. ^1^H-NMR (400 MHz, CDCl_3_) δ = 8.08 (*d*, *J* = 7.7 Hz, 1H), 7.62 (*s*, 1H), 7.40 (*t*, *J* = 7.8 Hz, 1H), 7.13 (*t*, *J* = 7.6 Hz, 1H), 6.89 (*d*, *J* = 7.9 Hz, 1H), 6.31 (*s*, 1H). (*Z*): ν_max_ (cm^−1^): 3220, 2212, 1720, 1618. ^1^H-NMR (400 MHz, CDCl_3_) δ = 7.72 (*d*, *J* = 7.7 Hz, 1H), 7.58 (*s*, 1H), 7.41 (*t*, *J* = 7.6 Hz, 1H), 7.04 (*t*, *J* = 7.6 Hz, 1H), 6.94 (*s*, 1H), 6.87 (*d*, *J* = 7.7 Hz, 1H). HRMS (ES) *m*/*z* calculated for C_10_H_6_N_2_O [M + H]^+^ 171.0558, found 171.0556.

*2-(5-Bromo-2-oxoindolin-3-ylidene)acetonitrile* (Table 4, **6b**). Yield: 26%. Orange solid; m.p.: 254–256 °C. ν_max_ (cm^−1^): 3193, 2213 (CN), 1721, 1605, 1455, 1445, 1300, 1219, 1115, 1059, 876, 821. ^1^H-NMR ^1^H (400 MHz, acetone-*d*_6_) δ (*E* + *Z*) = 9.96 (*s*, 1H, *E*), 9.92 (*s*, 1H, Z), 8.07 (*d*, *J* = 1.9 Hz, 1H, *E*), 7.93 (*d*, *J* = 1.9 Hz, 1H, *Z*), 7.62 (*dd*, *J* = 8.4, 2.0 Hz, 1H, *E*), 7.57 (*dd*, *J* = 8.3, 2.0 Hz, 1H, *Z*), 7.01 (*d*, *J* = 8.4 Hz, 1H, *E*), 6.96 (*d*, *J* = 8.4 Hz, 1H, *Z*), 6.82 (*s*, 1H, *Z*), 6.54 (*s*, 1H, *E*). HRMS (ES) *m*/*z* calculated for C_10_H_5_N_2_OBr [M + H]^+^ 248.9664, found 248.9661.

*2-(5-Chloro-2-oxoindolin-3-ylidene)acetonitrile* (Table 4, **6c**). Yield: 27%. Orange solid; m.p.: 241–243 °C. ν_max_ (cm^−1^): 3186, 2214 (CN), 1730, 1609, 1457, 1450, 1298, 1217, 1116, 1069, 822. ^1^H-NMR (400 MHz, acetone-*d*_6_) δ (*E* + *Z*) = 9.95 (*s*, 2H, *E* + *Z*), 7.93 (*d*, *J* = 2.1 Hz, 1H, *E*), 7.81 (*d*, *J* = 2.1 Hz, 1H, *Z*), 7.49 (*dd*, *J* = 8.4, 2.1 Hz, 1H, *E*), 7.43 (*dd*, *J* = 8.4, 2.2 Hz, 1H, *Z*), 7.06 (*d*, *J* = 8.5 Hz, 1H, *E*), 7.00 (*d*, *J* = 8.3 Hz, 1H, *Z*), 6.83 (*s*, 1H, *Z*), 6.55 (*s*, 1H, *E*). ^13^C-NMR (400 MHz, acetone-*d*_6_) δ (*E* + *Z*) = 166.52 (*E*), 165.26 (*Z*), 144.44 (*E*), 144.29 (*Z*), 143.98 (*E*), 143.10 (*Z*), 134.28 (*E*), 133.97 (*Z*), 128.03 (*E*), 127.91 (*Z*), 124.88 (*E*), 123.81 (*Z*), 123.57 (*Z*), 122.24 (*E*), 117.00 (*E*), 115.76 (*Z*), 113.33 (*E*), 112.97 (*Z*), 99.98 (*Z*), 99.93 (*E*). HRMS (ES) *m*/*z* calculated for C_10_H_5_ClN_2_O [M + Na]^+^ 226.9988, found 226.9989.

*2-(5-Nitro-2-oxoindolin-3-ylidene)acetonitrile* (Table 4, **6d**). Yield: 56%. Orange solid; m.p. > 300 °C. ν_max_ (cm^−1^): 3179, 2218 (CN), 1729, 1702, 1619, 1533, 1465, 1345, 1310, 1207, 1150, 1070, 844, 745. ^1^H-NMR (400 MHz, DMSO-*d*_6_) δ (*E* + *Z*) = 11.60 (*s*, 1H, *E*), 11.56 (*s*, 1H, *Z*), 8.72 (*d*, *J* = 1.7 Hz, 1H, *E*), 8.61 (*d*, *J* = 1.8 Hz, 1H, *Z*), 8.34 (*dd*, *J* = 8.7, 1.9 Hz, 1H, *E*), 8.28 (*dd*, *J* = 8.8, 1.9 Hz, 1H, *Z*), 7.34 (*s*, 1H, *Z*), 7.11 (*d*, *J* = 8.8 Hz, 1H, *E*), 7.04 (*d*, *J* = 8.7 Hz, 1H, *Z*). 6.80 (*s*, 1H, *E*). ^13^C-NMR (400 MHz, DMSO-*d*_6_) δ (*E* + *Z*) = 166.39 (*E*), 165.17 (*Z*), 150.20 (*E*), 148.84 (*Z*), 142.85 (*E*), 142.48 (*Z*), 142.36 (*Z*), 142.10 (*E*), 129.83 (*E*), 129.47 (*Z*), 121.37(*Z*), 119.82 (*E*), 119.28 (*Z*), 119.09 (*E*), 116.46 (*Z*), 115.40 (*E*), 111.25 (*E*), 110.82 (*Z*), 101.22(*E* + *Z*). HRMS (ES) *m*/*z* calculated for C_10_H_5_N_3_O_3_ [M + Na]^+^ 238.0228, found 238.0231.

## 4. Conclusions

This work described novel examples of the importance of the 1,2,3-dithiazole skeleton in chemistry. It also confirmed the utility of Appel’s salt in the conception of novel heterocyclic rings to access original potential bioactive compounds. The addition of Appel’s salt to 2-oxindoles could be performed efficiently and stereoselectively. The corresponding 3-substituted oxindole derivatives could be subjected to selective ring opening, giving fast access to diversely functionalized oxindoles at position 3. Thanks to these methods, we easily generated two families of compounds—3-(1,2,3-dithiazolylidene)indololin-2-ones (Table 2, **3a**–**d**) and 2-mercapto-2-(2-oxoindolin-3-ylidene)acetonitriles (Table 3, **4a**–**d**)—that could have interesting biological properties, such as protein kinase inhibition or antimicrobial properties. Optimization of the reaction conditions with triphenylphosphine should be performed in an effort to increase yields, and to learn more about stereoselectivity, since it seemed that most 2-(oxoindolin-3-ylidene)acetonitrile products (Table 4, **6b**–**6d**) were (E) isomers.

## Figures and Tables

**Table 1:** Synthesis of 3-(1,2,3-dithiazol-ylidene)indolin-2-ones (**3a**–**d**) from indolin-2-ones (**2a**–**d**) with Appel’s salt (1.1 equivalent).

Starting Material	R	Dithiazole 3	Yield (%) ^a^	Chemical Shift of H4 in ppm ^b^
**2a**	H	**3a**	96	8.19 (7.20) ^c^
**2b**	5-Br	**3b**	96	8.36 (7.36)
**2c**	5-Cl	**3c**	99	8.24 (7.20)
**2d**	5-NO_2_	**3d**	85	9.13 (8.07)

^a^ Isolated yield. ^b^ The chemical shift of the corresponding indolin-2-ones is given in brackets for comparison. ^c^ Chemical shift attributed to the H4 proton through comparison of one-dimensional (1D) NMR spectra of the dithiazole and the corresponding 2-oxindole.

**Table 2:** Yields of conversion of 3-(1,2,3-dithiazol-5-ylidene)indololin-2-ones (**3a**–**d**) into 2-mercapto-2-(2-oxoindolin-3-ylidene)acetonitriles (**4a**–**d**) and 2,2′-disulfanediylbis(2-(2-oxoindolin-3-ylidene)acetonitriles) (**5a**–**d**).

Dithiazole	R	Mercapto	Yield %	Yield %	Dimer	Yield %	Yield %
		Compound	NaH	CH_3_MgBr		NaH	CH_3_MgBr
**3a**	H	**4a**	49	33	**5a**	46	30
**3b**	5-Br	**4b**	66	25	**5b**	33	5
**3c**	5-Cl	**4c**	68	-	**5c**	30	-
**3d**	5-NO_2_	**4d**	88	-	**5d**	-	-

**Table 3:** ^1^H-NMR data δ of H4 (in ppm) of the mercaptoacetonitriles (**4a**–**4d**) and the disulfur dimers (**5a**–**5c**).

Mercapto	δ of H4 (in ppm)	Dimer	δ of H4 (in ppm)
**acetonitrile**			
**4a**	8.99	**5a**	8.10
**4b**	9.23	**5b**	9.08
**4c**	9.55	**5c**	8.86
**4d**	9.91	**5d**	-

**Table 4:** Yield of conversion of dithiazole (**3a**–**3d**) into 2-(2-oxindolil-3-ylidene)acetonitriles (**6a**–**6d**) with two equivalents of supported triphenylphosphine.

Compd.	R	Yield (%) ^a^	δ ppm (Z) Isomer	δ ppm (Z) Isomer	δ ppm (E) Isomer	δ ppm (E) Isomer	% (E) Isomer ^b^
**6**			H4	H-vinyl	H4	H-vinyl	
**6a**	H	88	7.72	6.94	8.08	6.31	80
**6b**	5-Br	26	7.93	6.82	8.07	6.54	80
**6c**	5-Cl	27	7.81	6.83	7.93	6.55	86
**6d**	5-NO_2_	56	8.61	7.34	8.72	6.80	83

^a^ Isolated yield of stereoisomer mixture. ^b^ Percentage determined from integration of NMR signals.
